# Genome-wide landscape of liver X receptor chromatin binding and gene regulation in human macrophages

**DOI:** 10.1186/1471-2164-13-50

**Published:** 2012-01-31

**Authors:** Petri Pehkonen, Lynn Welter-Stahl, Janine Diwo, Jussi Ryynänen, Anke Wienecke-Baldacchino, Sami Heikkinen, Eckardt Treuter, Knut R Steffensen, Carsten Carlberg

**Affiliations:** 1 School of Medicine, Institute of Biomedicine, University of Eastern Finland, FIN-70210 Kuopio, Finland; 2 Life Sciences Research Unit, University of Luxembourg, L-1511 Luxembourg, Luxembourg; 3 Department of Biosciences and Nutrition, Karolinska Institutet, S-14183 Huddinge, Sweden

## Abstract

**Background:**

The liver X receptors (LXRs) are oxysterol sensing nuclear receptors with multiple effects on metabolism and immune cells. However, the complete genome-wide cistrome of LXR in cells of human origin has not yet been provided.

**Results:**

We performed ChIP-seq in phorbol myristate acetate-differentiated THP-1 cells (macrophage-type) after stimulation with the potent synthetic LXR ligand T0901317 (T09). Microarray gene expression analysis was performed in the same cellular model. We identified 1357 genome-wide LXR locations (FDR < 1%), of which 526 were observed after T09 treatment. *De novo *analysis of LXR binding sequences identified a DR4-type element as the major motif. On mRNA level T09 up-regulated 1258 genes and repressed 455 genes. Our results show that LXR actions are focused on 112 genomic regions that contain up to 11 T09 target genes per region under the control of highly stringent LXR binding sites with individual constellations for each region. We could confirm that LXR controls lipid metabolism and transport and observed a strong association with apoptosis-related functions.

**Conclusions:**

This first report on genome-wide binding of LXR in a human cell line provides new insights into the transcriptional network of LXR and its target genes with their link to physiological processes, such as apoptosis.

The gene expression microarray and sequence data have been submitted collectively to the NCBI Gene Expression Omnibus http://www.ncbi.nlm.nih.gov/geo under accession number GSE28319.

## Background

The nuclear receptors liver X receptor (LXR) α and β (encoded by the genes *NR1H3 *and *NR1H2*) are transcription factors that act as sensors for oxidized cholesterol (oxysterols) [1-3]. High expression levels of LXRα in metabolic active tissues fit with the central role of the receptor in lipid metabolism, while LXRβ is more ubiquitously expressed [4]. Interestingly, both LXRs are found in various cells of the immune system such as macrophages, dendritic cells and lymphocytes, which suggests an important function in the innate and adaptive immune response [5-7]. In macrophages the accumulation of excess lipoprotein-derived cholesterol activates LXR and triggers the induction of a transcriptional program for cholesterol efflux, such as ATP-binding cassette transporter (ABC) A1 (*ABCA1*) and *ABCG1*, while in parallel the receptor transrepresses inflammatory genes, such as inducible nitric oxide synthase, interleukin 1β and monocyte chemotactic protein-1 [8-11].

Oxysterols and intermediates of the biosynthetic cholesterol pathway have been identified as the natural ligands for LXR, while T0901317 (T09) is a potent synthetic LXR agonist with an EC_50 _of about 50 nM [12,13]. LXRs bind to DNA as a heterodimer with the nuclear receptor retinoid X receptor (RXR) on direct repeats (DRs) of (A/G)GGTCA core binding motifs with four intervening nucleotides (DR4) [14]. These DR4-type response elements (REs) have been identified in the regulatory regions of a number of primary LXR target genes [15-17]. Recently, the first genome-wide views of LXR binding were obtained in a murine macrophage cell line [18] and in mouse liver [19]. In the murine macrophage study overexpressed biotin-tagged LXRβ was used for the chromatin immunoprecipitation (ChIP), followed by massive parallel sequencing (ChIP-Seq). *De novo *motif analysis identified DR4-type REs as the most highly enriched binding sequence, but only 6.3% of the LXR peaks contained such a DR4-type RE. Also motifs for the transcription factors PU.1 and AP-1 were co-enriched [18].

The ability of LXRs to integrate lipid metabolism and immune functions places them in an ideal position to tailor macrophage responses. Therefore, we performed a genome-wide analysis of T09-induced LXR binding in macrophage-type phorbol myristate acetate (PMA)-differentiated THP-1 human monocytic leukemia cells by ChIP-Seq using a highly specific anti-LXR antibody. In total we identified 1357 chromosomal LXR binding locations, of which 526 were observed after the T09 treatment. At the mRNA level the ligand induced 1258 genes and repressed 455 genes. We studied the LXR cistrome and identified LXR-enriched genomic regions as well as individual LXR target genes. Binding of LXR is focused on 112 genomic regions with individual constellations of binding sites and target genes. We could confirm that LXR controls functions related to lipid metabolism and observed a strong link to apoptosis. Therefore, this first report on genome-wide binding of LXR in a human cell line provides new insights into LXR target genes and their link to physiological processes, such as apoptosis.

## Results

### Genome-wide binding of LXR in a human macrophage-type cell line

THP-1 human monocytic leukemia cells were treated for 3 days with PMA to induce a human macrophage-type model and subsequently treated for 60 min with the potent synthetic LXR agonist T09 or vehicle DMSO. ChIP assays were performed using an antibody specific for LXRα and β (and non-specific IgG as negative control). The LXR antibody was successfully used in regular ChIP assays [20,21] and also applied in a very recent LXR ChIP-seq study in mouse liver [19]. Its specificity is further demonstrated in Western blot analysis (see additional file 1: **Figure S1**), where the antibody recognizes both LXRα and LXRβ protein in liver from wild type mice. LXRα is not recognized in LXRα^-/- ^mice, LXRβ is not recognized in LXRβ^-/- ^mice and both LXR bands disappear in the LXRαβ^-/- ^mice. Furthermore, the antibody recognizes both human LXRα and LXRβ when they are overexpressed in HeLa cells. This indicates that the antibody is specific to both mouse and human LXRα and LXRβ.

**Figure 1:**
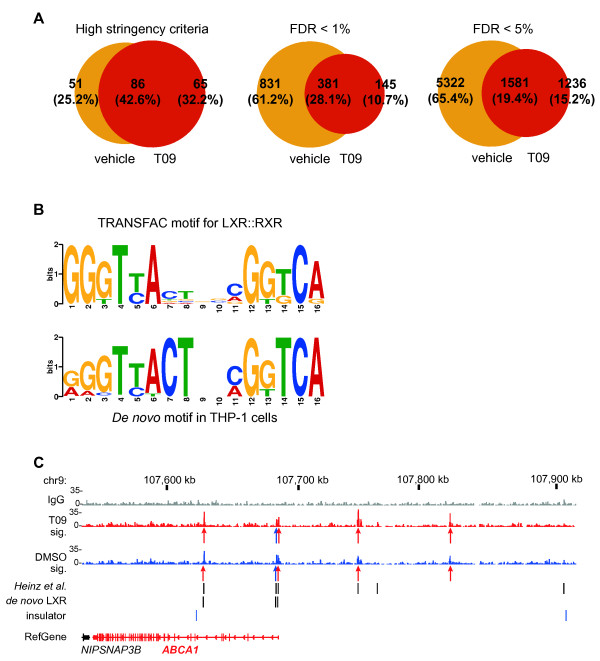
**Genome-wide LXR binding locations in a human macrophage-type cell line. A **Overlap between LXR binding locations in T09- and vehicle-treated human macrophage-type cells (THP-1 cells treated for 3 days with 20 nM PMA) under three different stringency criteria for ChIP-Seq peak selection. The high stringency criterion is comprised of the thresholds for FDR < 1%, FE > 4 and raw P-value < 10^-10^, while the two less stringent criteria were simply FDR < 1% and FDR < 5%. **B ***De novo *motif obtained using sequences for ChIP-Seq peaks with FDR < 1% (± 100 bp from peak summits) and the corresponding Transfac motif obtained from the public TOMTOM tool [25]. **C **Genomic region of the LXR target gene *ABCA1 *that is up-regulated (indicated by red color) by T09 treatment. Indicated are ChIP-Seq read alignment tracks for the IgG control (gray), the T09-treated sample (red) and the vehicle-treated sample (blue). Under each sample track the arrows indicate LXR binding locations of high stringency (red) or FDR < 1% (blue). Extra lanes indicate the location of LXR in homologous sequences of the mouse genome as previously published [18], *de novo *LXR binding sites within the peak area and insulator barrier regions identified via CTCF binding sites observed in CD4^+ ^T cells [32].

Purified chromatin samples were sequenced using a Solexa GAII platform. In order to detect genomic LXR binding locations, we used Bowtie software [22] for the read sequence alignment and the MACS program [23] for detection of statistically significant pileups of fragments when comparing to IgG. The number and overlap of detected LXR binding locations in and between the T09- and vehicle-treated cells are shown in Figure 1A (see additional file 2: **Table S1 **for all binding locations). Since the use of a single criterion for the selection of a representative peak set could be restrictive for the further analysis, we considered three criteria, each with different stringency.

The first high stringency criterion with false discovery rate (FDR) < 1%, fold enrichment (FE) > 4 and raw P-value < 10^-10 ^showed a higher number of peaks in the ligand-treated sample (151 peaks) than in the vehicle-treated sample (137 peaks), suggesting that the ligand slightly induces LXR binding among the high confidence binding locations. Interestingly, the peaks of both T09- and vehicle-treated samples were enriched to exon and intron regions, but ligand treatment clearly increased the density of LXR binding close to the transcription start site (TSS) (see additional file 3: **Figure S2**). Due to these very stringent criteria the total number of peaks is not very high (202 peak locations), but they have proportionally higher overlap between T09- and vehicle-treated samples (42.6%) than the two following criteria showing overlaps of only 28.1% and 19.4%.

The second stringency level using a single criterion of a FDR < 1% increased the number of LXR binding sites in the T09-treated sample to 526 peaks and even more (1212 peaks) in the vehicle-treated sample. In total, this represents 1357 LXR binding locations. Loosening the stringency to FDR < 5% further increases the total number of LXR binding locations in the vehicle-treated sample (6903 peaks) compared to the ligand treatment (2817 peaks). In general, ligand treatment seems to increase the number of high confidence peaks and to decrease the number of lower confidence peaks suggesting that liganded LXR is focusing on a fewer number of genomic binding sites of possibly increased importance.

We then applied the *de novo *motif detection tool MEME [24] on the sequences within ± 100 bp of the summits of the 1357 LXR peaks with a FDR < 1%. From the top 10 results obtained with the TOMTOM tool [25] in the T09-treated peaks, only one sequence motif resembled a known transcription factor binding site with similarity score E < 10^5^. This motif has a MEME score of E = 1.4 × 10^-12 ^and the TOMTOM tool indicated its high similarity to two DR4-type LXR consensus matrices reported in the Transfac database (M00766 with E = 4.95 × 10^-10 ^and M00647 with E = 1.60 × 10^-8^) (Figure 1B). As a major difference, the first two nucleotides of the spacer (positions 7 and 8) seem to form a consensus "CT" in our matrix, whereas in the best matching Transfac matrix these positions are not clearly enriched. The motifs reported by MEME of the vehicle-treated sample included several compositionally biased hits and none of them could be recognized by TOMTOM.

Further analysis of all 1357 peak sequences with FDR < 1% using the matrix screening function of the regulatory sequence analysis tools (RSAT) web server [26] showed that a site similar to the *de novo *derived DR4-type RE or its version with spacer positions 7 and 8 set equal to any nucleotide (see Methods for details) can be found in 7.4% (39 of 526 peaks) of the T09-treated peak set and in 7.2% (87 of 1212) of the vehicle-treated peak set with similarity P < 10^-4^. When the similarity threshold was reduced to P < 10^-3 ^the number of DR4-type REs could be increased to 42.6% and 41.3% in T09- and vehicle-treated samples, respectively (data not shown). Due to relatively low percentage of DR4-type REs, we screened the high stringency LXR peak sequences for the nuclear receptor half-site motif RGKTCA in a DR 0-6, everted repeat (ER) 0-12 and inverted repeat (IR) 0-6 arrangement. This resulted in enrichment for DR1-, DR4- and IR1-type REs in the T09-treated sample and DR4- and ER9-type REs in the vehicle-treated sample (see additional file 4: **Figure S3**). Direct LXR-RXR heterodimer binding to DR1- and ER9-type REs has not yet been found, but it had been reported on an IR1-type RE [27].

Screening of the LXR peak sequences with all 459 non-redundant JASPAR database matrices (JASPAR core, version 10/2009), without DR4-type LXR RE among them, resulted in the following five most frequent motifs in the T09-treated sample (with similarity P < 10^-4^): EWSR1-FLI1 (15.4%), KLF4 (13.7%), RREB1 (13.7%), SP1 (13.1%) and the DR1-type RE PPARG:RXR (12.0%). In the vehicle-treated sample these were EWSR1-FLI1 (17.6%), MYF (11.8%), SP1 (11.8%), RREB1 (11.7%) and SPIB (10.9%). Among the top motifs SPIB represents a purine-rich ETS-type motif, which is also recognized by the transcription factor PU.1, previously found in the LXR ChIP-Seq analysis from the murine macrophage cell line RAW264.7 [18]. The EWSR1-FLI1 matrix also represents a binding site for ETS-type transcription factors with various functions, such as cell cycle regulation and cell migration [28]. RREB1 is known to relate to RAS-mediated cell differentiation [29] and SP1 and KLF4 both represent binding sites for KLF-family proteins, where KLF4 is known to drive the differentiation of immune cells [30]. As the motif PPARG:RXR was the fifth most frequent in the T09-treated sample and the *PPARG *gene was also detected as up-regulated with a FC = 1.4 in our microarray at 4 h, we decided to search peaks with FDR < 1% for motifs PPARG::RXRA (DR1-type PPRE based on ChIP-Seq performed in mouse with identifier MA0065.2) and PPARG (Pal3-type PPRE based on Selex screening performed in human with identifier MA0066.1), available in JASPAR. These covered together 16.5% (87 of 526) of the T09-treated and 14.3% (173 of 1212) of the vehicle-treated peak set with a similarity P < 10^-4^. Interestingly, most of the peaks containing a PPARγ binding motif did not contain any DR4-type RE motif (79 of 87 peaks (90.8%) in the T09-treated and 162 of 173 peaks (93.6%) in the vehicle-treated peak set). These results indicate that some of the LXR peaks observed in ChIP-Seq data could be explained by an indirect DNA binding of LXR via other transcription factors, such as PPARγ, or by cooperative direct DNA binding of LXR together with some of the mentioned transcription factors.

A large part (42.6%) of the LXR binding locations in the high stringency set (Figure 1A) represent the well understood case of LXR being present on its genomic binding site both before and after the ligand treatment. This is illustrated at the genomic region of the well-known LXR target gene *ABCA1 *[31] showing four peaks from the stringent set and one additional from the FDR < 1% set (Figure 1C). This observation corroborates our previous report of LXR binding sites on the *ABCA1 *gene by regular ChIP assays [20]. Moreover, of the five sites observed at the *ABCA1 *gene, three contain DR4-type REs and four correspond to those detected previously in the first mouse LXR ChIP-Seq study [18]. All five peaks were also occupied by LXR in the absence of ligand, but after T09 treatment the intensity of LXR binding increased. Interestingly, the five LXR locations and the *ABCA1 *TSS are contained within the same block, which is flanked by CTCF binding sites (data from in CD4^+ ^T cells [32]) that are known as chromatin barrier insulators. This suggests that this genomic region displays the complete set of binding sites needed to understand the LXR regulation of the *ABCA1 *gene.

In summary, depending on chosen stringency criteria we detected in a human macrophage-type cell line between 202 and 8139 genomic LXR binding sites. The only identified *de novo *motif in the peak areas was a DR4-type RE, but matrix screening also identified binding sites for other transcription factors, such as PPARγ. The example of the *ABCA1 *locus indicates that our ChIP-Seq data can fully explain the LXR regulation of a T09 target gene.

### Spatial clusters of LXR binding locations and regulated genes

Next we studied the genome-wide clustering of LXR binding sites and the location of target genes within the clustered chromosomal regions with enrichment of binding locations. We stimulated macrophage-type PMA-treated THP-1 cells for 4 h with T09 or vehicle (DMSO), extracted total RNA and analyzed it on Illumina Human HT-12 v3 Expression BeadChip gene expression microarrays. Statistical analysis detected 1713 regulated genes (P < 0.01), out of which 1258 were up and 455 down-regulated (see additional file 5: **Table S2**). The response already after 4 h suggests that these genes are primary LXR target genes.

**Figure 2:**
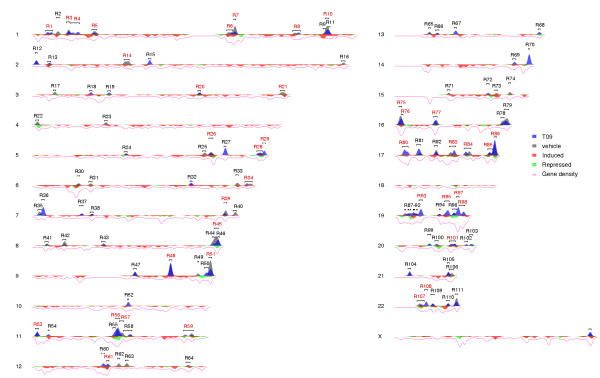
**Spatial genomic organization of LXR binding locations in relation to T09 target genes**. The 202 LXR locations of the high stringency peak set (Figure 1A) and the 1713 T09-regulated genes with adjusted P < 0.01 in 22 + X human chromosomes are visualized. A 1 Mb sliding window was used for the density graphs. These were further segmented, in order to detect chromosomal regions enriching LXR binding locations. One hundred twelve genomic regions were identified as hotspots for LXR actions, of which those with ≥ 2 peaks and ≥ 3 regulated genes are highlighted in red.

The genome-wide organization of the high stringency set of 202 LXR binding locations (Figure 1A) and of the 1713 T09-regulated genes in their physical context is shown in Figure 2 using window density based visualization. This visualization shows the densities of ChIP-Seq peaks and regulated genes across the genome within 1 Mb windows. The window densities are weighted by the FEs of ChIP-Seq peaks and the log_2 _fold changes (FC) of the differentially expressed (DE) genes, which emphasizes high peaks or extremely differentially expressed genes. Subsequent analysis of the window density data using segmentation (for details, see Methods) to detect the exact borders of the peak-enriched regions resulted in the indicated 112 distinct genomic areas. Regions with ≥ 2 peaks and ≥ 3 T09-regulated genes are highlighted in red and listed in Table 1 (the complete list of all regions with extended information is summarized in additional file 6: **Table S3**).

**Table 1:** Genome-wide hotspots of LXR action

Region number	Region coordinates		LXR sites		Regulated genes		Regulated gene names
	Chr	Location	T09	vehicle	Up	Down	
R1	1	8412456-11763591	2	1	3	2	*RP3-477M7.4, KIF1B, EXOSC10, RERE, ENO1*
R3	1	23630263-26071982	2	2	3	1	*RPL11, PNRC2, CLIC4, HNRNPR*
R4	1	27018838-33511169	2	0	3	3	*YTHDF2, SPOCD1, KHDRBS1, SLC9A1, PUM1, SNRNP40*
R5	1	43282794-47844512	2	2	6	3	*ERMAP, MED8, POMGNT1, KIAA0494, STIL, CMPK1, GPBP1L1, TMEM69, MKNK1*
R6	1	150237798-154193105	1	2	8	2	*MRPS21, MCL1, ENSA, GABPB2, S100A9, S100A8, RPS27, C1orf43, APH1A, PSMD4*
R7	1	154934773-156706753	1	3	4	2	*GON4L, KIAA0907, SLC25A44, CCT3, SHC1, C1orf66*
R8	1	201798268-207228338	1	2	5	2	*IPO9, UBE2T, KDM5B, TMEM183A, RBBP5, ADIPOR1, SOX13*
R10	1	226626264-229694443	4	4	4	0	*RP11-118H4.1, FTHL2, SPHAR, ABCB10*
R14	2	69240275-73460367	0	2	5	2	*ANTXR1, GFPT1, SNRNP27, MCEE, C2orf7, TEX261, RAB11FIP5*
R20	3	127317065-129041381	1	1	1	2	*CNBP, MCM2, C3orf37*
R21	3	193585240-196669469	0	2	4	2	*TM4SF19, AC092933.3, PAK2, NCBP2, TNK2, AC024937.4*
R26	5	137273648-140086267	2	2	6	5	*FAM13B, BRD8, ETF1, MATR3, PURA, C5orf53, EGR1, C5orf32, PFDN1, HARS, ZMAT2*
R28	5	175818220-179780388	2	1	2	5	*RP11-889L3.1, RNF130, PRELID1, HNRNPAB, RUFY1, CANX, GFPT2*
R29	5	178977558-180671711	2	0	1	3	*RNF130, RUFY1, CANX, GFPT2*
R34	6	166778406-170893749	1	2	4	0	*BRP44L, CCR6, PSMB1, PDCD2*
R39	7	150069060-151361890	1	2	2	2	*GIMAP8, FASTK, GIMAP5, AGAP3*
R45	8	143354160-145736580	4	5	0	4	*TOP1MT, PUF60, BOP1, MFSD3*
R48	9	106856540-108403400	4	4	3	0	*SMC2, ABCA1, FKTN*
R51	9	138541640-140484943	7	9	1	2	*EDF1, PMPCA, ZMYND19*
R53	11	308106-911658	1	2	1	2	*RPLP2, IFITM2, TMEM80*
R56	11	63448921-66445276	5	4	2	8	*TRMT112, PRDX5, RTN3, GPR137, DPF2, SSSCA1, FIBP, DPP3, RBM4, RBM4B*
R57	11	66247879-70053509	1	2	1	4	*FADD, DPP3, RBM4, RBM4B, SUV420H1*
R59	11	118230301-126138751	1	2	5	6	*UBE4A, AP000926.1, TMEM218, EI24, RPUSD4, ATP5L, ARCN1, HINFP, SLC37A2, FAM118B, SRPR*
R61	12	56229243-57628892	2	1	5	4	*AC034102.1, CNPY2, PTGES3, NACA, PRIM1, MMP19, RAB5B, RNF41,CS*
R75	16	108057-2759032	1	4	4	3	*STUB1, FAHD1, NDUFB10, PDPK1, RHBDF1, AC141586.5, KCTD5*
R76	16	2009518-3072385	3	1	3	2	*NDUFB10, PDPK1, TNFRSF12A, AC141586.5, KCTD5*
R77	16	28616902-31394319	3	3	2	2	*SULT1A1, VKORC1, QPRT, ITGAX*
R80	17	3572089-7418491	3	0	5	1	*TMEM93, ITGAE, MIS12, TXNDC17, ZBTB4, ACADVL*
R83	17	41843489-47502285	2	1	7	4	*DUSP3, GJC1, KIAA1267, KPNB1, PNPO, ZNF652, AC004797.1, NMT1, NSF, CDK5RAP3, NFE2L1*
R84	17	55015562-59147703	2	2	3	4	*COIL, SRSF1, AC005884.1, SUPT4H1, TRIM37, DHX40, TMEM49*
R85	17	73512608-76377808	2	1	3	3	*C17orf95, SRSF2, BIRC5, TSEN54, RHBDF2, SEC14L1*
R86	17	78863340-80685893	5	4	4	5	*ARL16, THOC4, SLC16A3, FN3KRP, AC127496.3, ACTG1, HGS, NARF, WDR45L*
R93	19	17416476-19617122	3	0	2	2	*PDE4C, C19orf50, MRPL34, RFXANK*
R95	19	39109721-42789931	3	1	6	2	*EIF3K, RPS16, EID2B, PSMC4, HNRNPUL1, RPS19, MRPS12, SERTAD3*
R97	19	49298321-51303301	3	0	0	3	*BCAT2, BAX, VRK3*
R98	19	52359054-56499996	2	1	4	1	*ZNF577, ZNF83, LILRB3, NLRP8, RPS9*
R101	20	42939823-48809213	2	2	6	3	*YWHAB, DNTTIP1, UBE2C, NCOA3, B4GALT5, RNF114, SERINC3, TMEM189-UBE2V1, CEBPB*
R107	22	17618400-22901769	1	1	4	7	*BID, PI4KA, RANBP1, AC002472.8, CECR5, ATP6V1E1, BCL2L13, COMT, C22orf25, XXbac-B33L19.3, PRAME*
R108	22	22890122-25024973	2	0	1	2	*MIF, PRAME, GGT1*

The genomic regions that contain two or more LXR binding sites and three or more T09-regulated genes (red in Figure 2). The locations of each region, the number of LXR peaks and the number and name of the regulated genes are indicated (for a full summary of all regions and additional statistics see additional file 6: **Table S3**).

**Figure 3:**
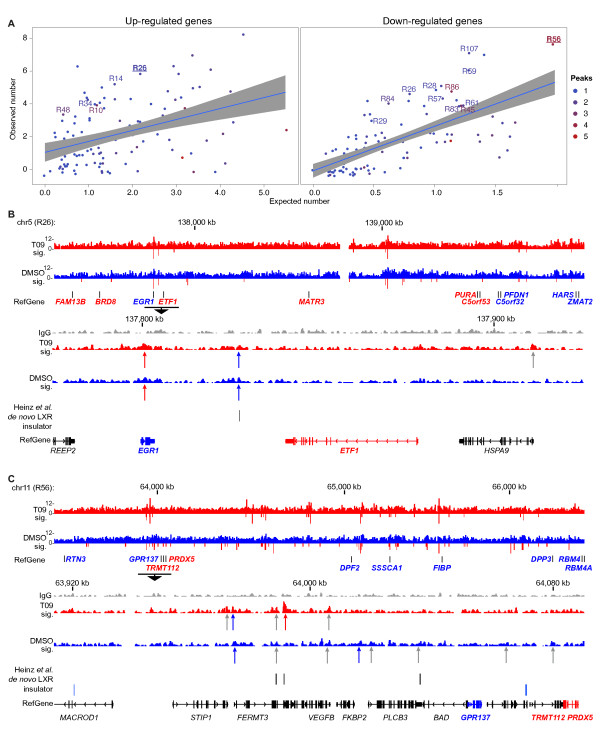
**LXR peak regions enriched in DE genes. A **Expected versus observed analysis of up- and down-regulated genes within the 112 LXR peak regions. The linear regression line is shown in blue and the 95% confidence interval as grey area. Each data point represents one region and is color-coded related to the number of LXR peaks it contains. Regions with a significantly (P < 0.05) enriched number of T09 target genes and at least two stringent LXR peaks are named. **B **Example of region R26. In the region overview only DE genes are shown (red = up-regulated, blue = down-regulated), while in the detailed view also the not-regulated genes are indicated in black. Under each sample track the arrows indicate LXR binding locations of high stringency (red), FDR < 1% (blue) or FDR < 5% (grey). Extra lanes indicate the location of LXR in homologous sequences of the mouse genome as previously published [18], *de novo *LXR binding sites within the peak area and insulator barrier regions identified via CTCF binding sites observed in CD4^+ ^T cells [32]. **C **Example of region R56.

Generally, the distribution of LXR binding locations in the genome-wide view (Figure 2) correlates with the density of all genes (*r *= 0.55) and slightly with the density of differentially expressed genes (*r *= 0.32), but the number of LXR peaks in the 112 hotspots does not correlate with the proportion of DE genes in these regions nor with the density of DE genes in these regions (see additional file 7: **Figure S4**). In a more detailed view, we correlated each of the 112 hotspot regions with the number of measured up-regulated (Figure 3A, left panel) and down-regulated (Figure 3A, right panel) genes with the number of expected up- or down-regulated genes. Using a binomial test with threshold P *<*0.05 to indicate statistical significance, we identified in both groups several regions that contain an unexpected high number of DE genes. Region R26 in chromosome 5 (Figure 3B) and region R56 on chromosome 11 (Figure 3C) are examples of this type of analysis. Region R26 contains six up- and five down-regulated genes and two high stringency LXR locations present in both T09- and vehicle-treated samples. The high stringency LXR location close to the T09 target genes *EGR1 *(early growth response protein) and *ETF1 *(eukaryotic translation termination factor 1) is shown in higher resolution (Figure 3B). Region R56 contains eight down- and two up-regulated genes and five high stringency LXR locations in the T09 sample and four in the DMSO sample, two of which are overlapping. Here the region upstream of the T09 target genes *GPR137 *(G protein-couple receptor 137), *TRMT112 *(tRNA methyltransferase 11-2 homolog) and *PRDX5 *(peroxiredoxin 5) is shown in higher resolution (Figure 3C).

However, there are also several differences in the location of LXR binding sites and T09 target genes. One of the most obvious differences is the differential expression of several genes on the X chromosome (Figure 2). With the exception of region R112 located at the 3' end of the X chromosome no LXR binding location in neither the high stringent nor in the FDR < 1% peak set could be detected. These genes without any LXR binding locations nearby may either represent effects of T09 that are not mediated by LXR, secondary targets of LXR or effects that are not visible in the ChIP-Seq dataset at the investigated time point. It should be noted that T09 has been shown to be also a ligand of other nuclear receptor superfamily members, such as farnesoid X receptor [33], pregnane X receptor [34], retinoid acid receptor-related orphan receptor [35] and androgen receptor [36]. However, based on our microarray analysis the genes encoding for farnesoid X receptor and pregnane X receptor, *NR1H4 *and *NR1I2*, are not expressed in PMA-differentiated THP-1 cells used in this study.

Taken together, the genome-wide correlation of the 202 high stringency LXR locations with 1258 up-regulated and 455 down-regulated T09 target genes indicated 112 genomic hotspots for the actions of LXR. Each of these hotspot regions provides a genomic scenario, where up to seven LXR binding sites may explain the regulation of up to eleven T09 target genes.

### Identification of direct LXR target genes

**Figure 4:**
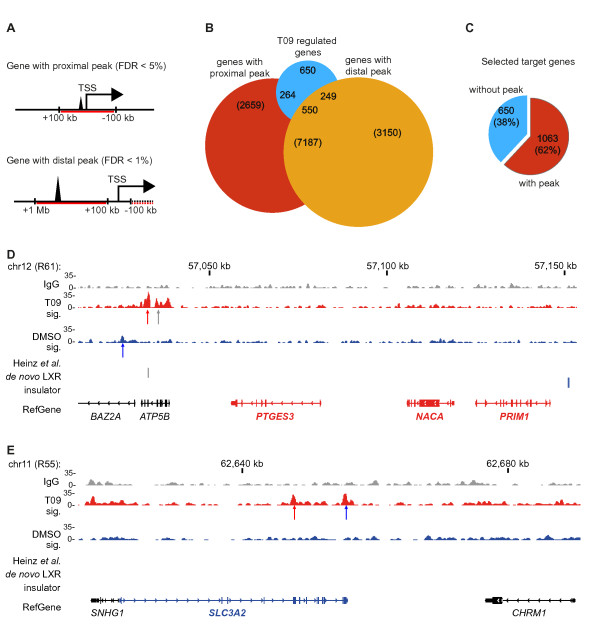
**Filtering for direct LXR target genes. A **Selection of target genes is based on LXR peaks with FDR < 5% within close (± 100 kb) or with FDR < 1% within distant (± 0.1-1 Mb) regions from the T09 target gene TSS. **B **Numbers of all and DE genes (corrected P < 0.01) with a LXR peak in the close or distant interval. **C **Selection of target genes based on the close and distant intervals and peak sets with FDR < 5% and FDR < 1% stringencies, respectively, results in 1063 LXR target genes (62%) among the all 1713 DE genes. **D **The locus of the most up-regulated gene *NACA *(based on the genes listed in Table 2) in region R61. Under each sample track the arrows indicate LXR binding locations of high stringency (red), FDR < 1% (blue) or FDR < 5% (grey). Extra lanes indicate the location of LXR in homologous sequences of the mouse genome as previously published [18], *de novo *LXR binding sites within the peak area and insulator barrier regions identified via CTCF binding sites observed in CD4^+ ^T cells [32]. **E **The locus of the most down-regulated gene *SLC3A2 *in region R55.

In order to discriminate those T09 target genes that are directly regulated by LXR compared to indirect LXR targets or no LXR targets at all, we examined the co-location of LXR ChIP-Seq peaks with the TSS of DE genes within proximal (0 to ± 100 kb) and distal (± 0.1 to ± 1 Mb) regions of peak summits (Figure 4A). For the proximal regions we used the looser criterion with FDR < 5%, whereas for the distal region we used the more stringent criterion FDR < 1%. From the 1713 DE T09 target genes 814 showed a LXR site within their proximal region and 799 a LXR peak within their distal region, 550 of which are overlapping (Figure 4B). This selection filtered out 38% (of which 85% were up-regulated) of the T09-regulated genes resulting in 1063, 706 up-regulated and 357 down-regulated, more probable direct LXR target genes (Figure 4C).

The 1063 genes with a LXR peak within 1 Mb of their TSS are marked in the list of all T09 target genes (see additional file 5: **Table S2**) and those that show an adjusted P *<*0.001 for differential expression and a proximal T09-induced LXR binding site are summarized in Table 2. From this selection we show the locus of the most up-regulated genes, *NACA *(nascent polypeptide-associated complex alpha subunit), flanked by the *PTGES3 *(prostaglandin E synthase 3) and *PRIM1 *(primase, DNA, polypeptide 1), which together form the core of region R61 (Figure 4D). Another exemplified locus is the most down-regulated gene, *SLC3A2 *(solute carrier family 3A2), located in region R55 (Figure 4E). In both examples there are dominant T09-induced tandem LXR peaks controlling the respective region, i.e. both the up-regulated (*PTGES3*, *NACA *and *PRIM1) *and the down-regulated (*SLC3A2) *genes appear to follow the same mechanism of a T09-induced LXR binding. In addition, we made a comparison with the LXR locations found previously in mouse macrophages [18] in a distance of 100 kb from the target gene TSS regions (see additional file 5: **Table S2**). Among all 1713 differentially expressed human genes only 7.9% (136 of 1713) showed LXR binding in the corresponding mouse region. Furthermore, among the 1063 selected target genes only 9.9% (105 of 1063) contained an LXR peak in the corresponding genomic region in mouse macrophages. Finally, from the human genes with proximal LXR peaks (Table 2) 25.8% (8 of 31) of their mouse homolog also showed LXR binding.

**Table 2:** LXR target genes with peaks in proximal region

Gene expression data					Peak within ± 100 kb			Peak within 100 kb-1 Mb	
**Chr**	**Start**	**P-value**	**Gene**	**FC**	**T09**	**vehicle**	**Heinz et al**.	**T09**	**vehicle**

12	57094565	8.29E-05	*NACA*	5.63	S	L		S	S
15	69745123	1.88E-04	*RPLP1*	5.19	M	L		L	M
2	47272677	2.28E-04	*CALM2*	4.20	M	M	X	L	M
12	53689235	1.57E-04	*PFDN5*	3.59	S	M		L	L
5	177482390	9.93E-04	*LOC653314*	3.20	M	L		S	S
22	20103461	1.14E-04	*RANBP1*	3.17	S	M		M	M
9	107543283	4.46E-04	*ABCA1*	2.94	S	S	X	S	S
21	43619799	8.29E-05	*ABCG1*	2.87	S	S	X	L	M
11	9681985	3.86E-04	*LOC731640*	2.79	S			L	M
4	154073494	3.16E-04	*TRIM2*	2.75	M	M		M	M
11	64084167	6.49E-04	*HSPC152*	2.71	S	M		S	S
16	2009519	5.19E-04	*NDUFB10*	2.70	M	L		S	S
1	40306708	1.12E-04	*TRIT1*	2.44	M	M		M	M
19	41768391	4.25E-04	*HNRPUL1*	2.37	S		X	S	M
1	107599301	9.61E-05	*PRMT6*	2.25	M	M			
16	50352941	1.44E-04	*BRD7*	2.20	M	M	X		L
9	128024073	5.65E-04	*GAPVD1*	2.02	M	M		M	M
6	16129356	1.63E-04	*MYLIP*	2.00	M		X		
1	156163730	2.09E-04	*SLC25A44*	1.97	S	S		M	S
1	228823162	5.41E-04	*FTHL2*	1.92	S	S		S	S
5	137841784	7.92E-04	*ETF1*	1.91	S	S		L	M
11	9685628	5.87E-04	*SWAP70*	1.86	S			L	M
19	12907634	5.28E-04	*PRDX2*	1.84	S	L	X	L	S
12	57057127	4.78E-04	*PTGES3*	1.76	S	L		S	S
3	186507669	5.41E-04	*RFC4*	1.73	M	M	X	L	L
17	7362685	6.20E-04	*ZBTB4*	1.67	S			L	
1	109852192	7.41E-04	*SORT1*	0.60	M	M		M	M
5	177631508	7.92E-04	*HNRNPAB*	0.59	S	S		M	M
9	137208944	8.64E-04	*RXRA*	0.56	M	M		S	M
3	57557090	9.67E-04	*ARF4*	0.55	S			L	M
11	62623518	1.88E-04	*SLC3A2*	0.43	S	L		S	S

Only genes with a T09 peak with FDR < 1% within ± 100 kb from their TSS are displayed (the full list of LXR target genes is available in additional file 5: **Table S2**). Symbols for peaks are as follows: S = stringent peak with FDR < 1%, FE > 4 and P < 10^-10^; M = peak with FDR < 1%; L = peak with FDR < 5%; X = any peak.

**Figure 5:**
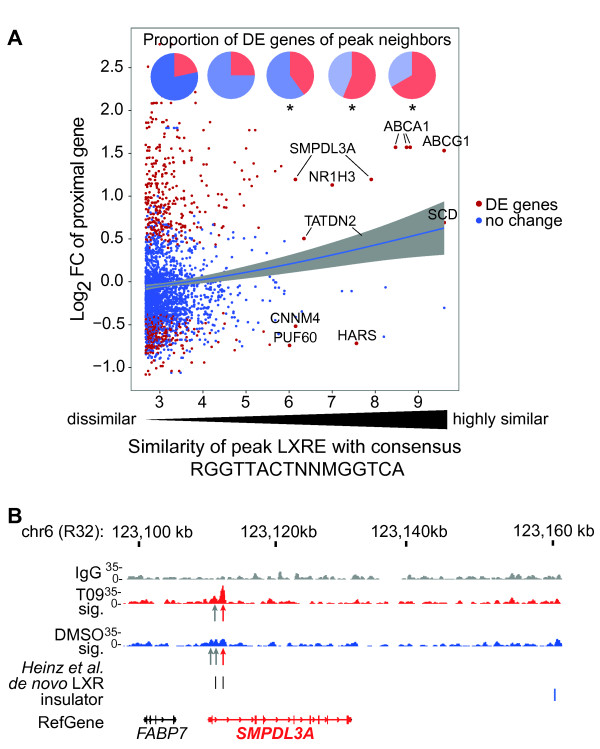
**LXR target genes with DR4-type REs. A **Analysis of LXR peak versus T09 target gene pairs by relating the log_2 _FC of target genes with the similarity of the DR4-type REs within the closest LXR peak (± 100 kb limit). For each peak, the gene with the smallest expression P-value in that region is represented. Similarity is indicated as -log_10 _of the P-value obtained from the RSAT motif screening tool [26]. Pie diagrams on the top show the proportions of differentially expressed gene-peak pairs among the all gene-peak pairs within the similarity intervals 3-4.5, 4.5-6, 6-7.5, 7.5-9 and > 9. In the DR4-type RE consensus sequence R = A or G, M = A or C, and N any nucleotide. **B **Example region of the T09 target gene *SMPDL3A *containing two DR4-type REs close to its TSS. Under each sample track the arrows indicate LXR binding locations of high stringency (red) or FDR < 5% (grey). Extra lanes indicate the location of LXR in homologous sequences of the mouse genome as previously published [18], *de novo *LXR binding sites within the peak area and insulator barrier regions identified via CTCF binding sites observed in CD4^+ ^T cells [32].

Next we compared altered expression of LXR target genes to the occurrence of DR4-type REs within the proximal LXR peaks (Figure 4A). Although DR4-type REs with very high similarity scores are not very common (Figure 1B), they seem to be enriched to the peaks in the vicinity of DE genes, especially when located nearby up-regulated genes (Figure 5A). This is consistent with the principal activation mechanism of LXR. The six up-regulated genes *ABCA1*, *ABCG1*, *SMPDL3A *(sphingomyelin phosphodiesterase, acid-like 3A), *NR1H3*, *SCD *(stearoyl-CoA desaturase) and *TATDN2 *(TatD DNase domain containing 2) and the three down-regulated genes *CNNM4 *(cyclin M4), *HARS *(histidyl-tRNA synthetase) and *PUF60 *(poly-U binding splicing factor 60 KDa) have LXR binding location with a motif highly similar (P < 10^-6^) to a DR4-type RE. The *ABCA1 *gene is one of the best-known LXR target genes and contains even three DR4-type REs within its regulatory region (Figure 1C). Another example, the *SMPDL3A *gene, carries two DR4-type REs within an LXR twin peak very close to its TSS (Figure 5B). Using quantitative real-time PCR (qPCR) and RNA from independently performed stimulation experiments of PMA-differentiated THP-1 cells with the synthetic LXR ligands T09 and GW3965 (GW), we validated nine representative LXR targets genes: the known targets *ABCA1*, *ABCG1*, *MYLIP *(myosin regulatory light chain interact), *NR1H3 *and *SCD *and the novel targets *PPARG*, *SMPDL3A*, *ADM *(adrenomedullin) and *ACSL3 *(acyl-CoA synthetase long-chain family member 3) (see additional file 8: **Figure S5**). After 4 h of ligand treatment all nine genes were significantly up-regulated by both LXR ligands.

In summary, 1063 of the 1713 T09 responding genes are probable direct targets of LXR, since 77% of them have at least one LXR binding site within 100 kb of their TSS and the further 23% show at least one LXR peak within 1 Mb distance. Interestingly, highly regulated LXR target genes have a higher probability to contain a DR4-type REs within the LXR peak associated with these genes.

### Association of functions to LXR target genes

In order to gain further insight into the possible common functions of the 1063 presumed direct LXR target genes identified, we performed a functional annotation analysis using the DAVID tool [37] and Gene Ontology (GO) biological process terms. This resulted in 78 GO terms with FDR < 5% mostly related to general themes, such as metabolism, translation and RNA processing (see additional file 9: **Table S4**). Among these, 71 genes are related to regulation of programmed cell death (rank 42) and 73 genes to regulation of apoptosis (rank 53). Interestingly, the immune-related terms are not enriched significantly, as the best ranking immune-related term "somatic diversification of immune receptors via germline recombination" is only related to five LXR target genes with FDR = 57.3%. We also analyzed separately a more specific gene list, a subset of 1063 genes, with P < 0.001 for differential expression (Table 3). Also here we mostly found more general functions, such as translation and RNA processing, and only two terms related to lipid handling. None of the terms were directly related to immune function but the two terms "cellular response to stress" and "response to virus" were detected. Separate analysis of genes with different binding profiles of LXR in their vicinity (being present either only in T09 samples, DMSO samples or both) did not show any significant enrichment of additional pathways (data not shown). This suggests a homogeneous function of LXR targets independent of possible different regulatory mechanisms.

**Table 3:** Enrichment analysis of high confidence LXR target genes

Term	Count	%	P	FE	FDR
translation	16	6.56	9.14E-05	3.34	0.15
lipid biosynthetic process	15	6.15	2.48E-04	3.21	0.41
cellular response to stress	20	8.20	5.40E-04	2.44	0.89
RNA splicing	13	5.33	8.58E-04	3.16	1.41
response to virus	8	3.28	1.00E-03	5.07	1.64
positive regulation of gene-specific transcription	7	2.87	1.59E-03	5.55	2.60
RNA splicing, via trans-esterification reactions	9	3.69	1.68E-03	4.06	2.75
nuclear mRNA splicing, via spliceosome	9	3.69	1.68E-03	4.06	2.75
RNA splicing, via trans-esterification reactions with bulged adenosine as nucleophile	9	3.69	1.68E-03	4.06	2.75
RNA processing	18	7.38	2.41E-03	2.27	3.90
negative regulation of cholesterol storage	3	1.23	2.98E-03	34.51	4.82

The analysis was performed using the DAVID tool [37] for the specific set of LXR target genes with P < 0.001 for differential expression. GO biological process terms with FDR < 5% are shown. Columns indicate the GO-term, its count and percentage in the target gene list, as well as the P-value, FE and FDR for the enrichment.

**Figure 6:**
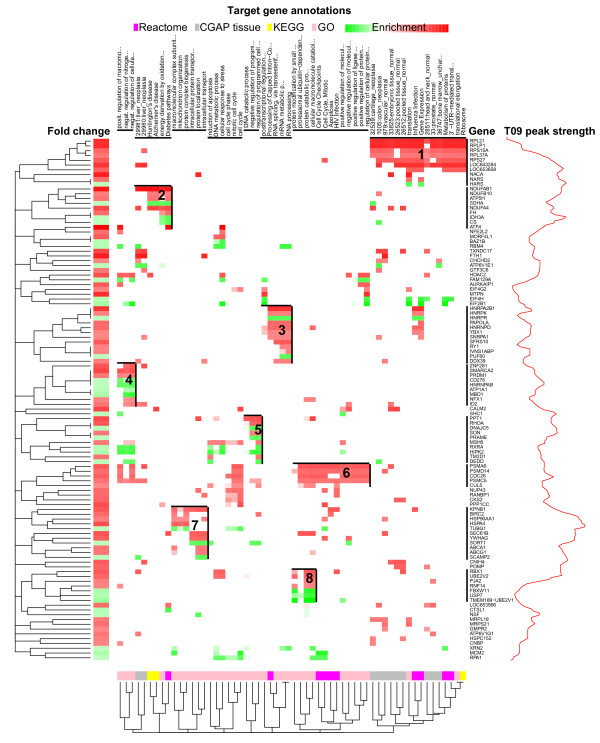
**Co-functional modules of direct LXR target genes**. Associations of LXR target genes with the annotations from Reactome, CGAP tissue EST expression, KEGG and GO databases clustered and visualized using heatmap.2 function from R-package gplots [38]. The y-axis columns indicate the annotations and x-axis rows the associated genes. Each association, depicted as a cell of the heat map, has been weighted using the multiplication of log_2 _FC of a gene (row) and -log_10 _P-value of annotation (column) enrichment highlighting the most important associations. Red and green color scales are used for up- and down-regulated genes, respectively. Both columns and rows have been clustered using agglomerative hierarchical clustering with asymmetric binary distance measure. The eight gene and annotation clusters cover the majority of the gene set. Indicated is also the LXR peak strength density graph, which summarizes the peak heights over the genes on the x-axis. For each gene the peak strength has been calculated as a sum of the log_2 _FEs for the highest peak from the close (± 100 kb) and distant region (± 0.1 to ± 1 Mb).

In order to detect the non-redundant sets of genes and associated biological themes, we performed a clustering analysis using enriched gene categories from the databases GO, Kyoto Encyclopedia of Genes and Genomes (KEGG), Reactome and CGAP tissue EST expression for the 1063 true LXR target genes. In order to ease the later inspection of results, we limited the number of genes used in the clustering analysis to the 150 most up-regulated and the 76 most down-regulated, preserving the same ratio as between all 706 and 357 up- and down-regulated LXR target genes. For the annotations, we used all that were enriched with FDR < 50%, corresponding to the raw P ≤ 0.036 in the GO enrichment analysis. We were able to use this loose criterion and obtain adequate data, because the clustering is robust to false positive annotations. The genes and associated annotations were clustered and visualized in parallel using the heatmap.2 function available in the R-package gplots [38]. Agglomerative hierarchical clustering with an asymmetric binary distance measure was used by treating each association between a gene and an annotation as 1 and a lack of association as 0. This resulted in eight visually homogeneous clusters of associated genes and annotations (Figure 6): translation-related genes (cluster 1), oxidation- and diabetes-related genes expressed in liver (cluster 2), mRNA processing-related genes (cluster 3), nitrogen metabolism-related genes (cluster 4), programmed cell death regulation-related genes (cluster 5), ubiquitin system genes with relation to cell cycle and apoptosis (cluster 6), genes related to intracellular transport including cholesterol transport (cluster 7) and general ubiquitin system-related genes (cluster 8).

Taken together, functional annotation analysis of the 1063 true LXR target genes using GO, KEGG, Reactome and CGAP tissue EST expression databases resulted in eight clusters, in which the functions apoptosis and lipid transport are found, but no link to immune functions were observed.

## Discussion

This study provides the first genome-wide view of LXR binding patterns in a human cell line using ChIP-Seq assays. We performed this study in PMA-differentiated THP-1 cells, a macrophage-type cellular system, which increases our understanding not only of the well-known role of LXR in lipid metabolism and transport, but also the receptor's assumed role in innate immunity and other physiological processes.

The highly stringent analysis of the ChIP-Seq peaks obtained from PMA-treated THP-1 cells after a 60 min stimulation with the synthetic LXR ligand T09 or its vehicle DMSO yielded a surprisingly low number of 202 genome-wide binding locations of the receptor. However, from the 151 LXR sites observed in the presence of T09, 57% (86) are also bound in the absence of ligand. This supports the canonical model for nuclear receptor binding being valid for most members of the superfamily [39]. According to this model the receptor binds genomic DNA already in the absence of ligand, probably in a complex with co-repressor and histone deacetylase proteins, and locally represses the chromatin structure. The addition of ligand induces a conformational change in the ligand-binding domain of the receptor, which then leads to dissociation of the co-repressor proteins and the recruitment of co-activators that open chromatin structure. Alternatively, co-activators act as mediators building a bridge to the basal transcription machinery, which leads to the activation of RNA polymerase II and gene transcription. According to the data presented here, this model seems to apply for a number of known LXR binding sites close to target genes, such as *ABCA1*.

A lower stringency in the detection of LXR ChIP-Seq peaks increased the number of sites substantially, to a total of 1357 (FDR < 1%) or even 8139 (FDR < 5%). However, with this increase of putative LXR binding sites the percentage of overlapping sites reduced to 28.1 and 19.4%, respectively, while the percentage of apparent binding sites in the absence of ligand increased to 61.2 and 65.4%, respectively. We assume that most of the latter sites are not genomic locations from which LXR initiates gene activation, but rather unspecific contact points of the genome with no functional impact. However, the tendency that ligand stimulation reduces the number of sites to less than half (from 1212 to 526 and from 6903 to 2817) suggests that in its ligand-activated state LXR makes a more focused selection of genomic targets.

Surprisingly, only 7.3% of the LXR peak summit sequences contain a DR4-type RE, which, based on *in vitro *studies, is the only high affinity DNA binding site for LXR-RXR heterodimers. This fits with the observations of the two presently published mouse LXR ChIP-Seq studies, where only 6.3% [18] or up to 8% [19] of the LXR peaks contained a DR4-type RE. In a very recent ChIP-on-chip report, 2035 LXRβ-RXR binding sites were identified within promoter regions of normal human epidermal keratinocytes [40]. Although 1666 (82%) of these rather large genomic fragments contain a kind of DR4-type RE, only 142 (7.0%) of them are highly scored, resulting in a very similar percentage as in our human and the mouse ChIP-Seq studies. For comparison, in our ChIP-Seq study on genomic vitamin D receptor (VDR) locations in undifferentiated THP-1 cells [41], we found only 31% of the VDR peak summits containing a VDR-specific DR3-type RE. We obtained the same result also by a re-analysis of VDR ChIP-Seq data from human lymphoblast cell lines [42,43]. These observations indicate that either the binding specificity of LXR (and VDR) is in the chromatin context far different than in *in vitro *assays (maybe due to cooperative binding with other transcription factors, such as PPARγ) or LXR is not directly contacting DNA, but sits "piggyback" on another DNA-binding transcription factor. Interestingly, in our T09 microarray the *PPARG *gene is 1.4-fold up-regulated, a value that was also confirmed by qPCR with two different LXR ligands. In parallel, we found for 16.5% of all LXR peaks in the T09-treated sample a PPARγ binding site. This suggests that PPARγ may be involved in the regulation of a subset of the LXR target genes. Boergesen *et al. *reported very recently that in mouse liver many genomic binding sites of LXR are also bound by PPARα, which is the predominant PPAR subtype in liver [19]. They suggest that PPARα helps LXR in recognizing its preferred genomic locations, a concept that may apply also for PPARγ in macrophages. Furthermore, Boergesen *et al. *also found a higher percentage of DR1-type REs below their LXR ChIP-Seq peaks than DR4-type REs, but in sum this can explain only less than 25% of all LXR locations. Therefore, they assume that both LXR and PPAR are far more promiscuous in the recognition of their binding sites as suggested by *in vitro *studies.

Nevertheless, we found DR4-type REs as functional LXR binding sites in a number of prominent and highly ligand-responsive LXR target genes, such as *ABCA1*, *ABCG1*, *NR1H3 *and *SCD*. Moreover, in a reasonable number of gene regulatory scenarios two or more genomic LXR binding sites seem to work together in the regulation of a gene cluster (for example, *NACA*, *GPR137 *and their respective neighboring genes) or individual genes (for example, *ABCA1*, *SLC3A2 *and *SMPDL3A*). These multiple RE arrangements confirm observations from single gene analyses with other nuclear receptors, such as the VDR [44-46].

More important than the actual number of individual LXR binding sites may be their spatial clustering along the genome. We identified 112 genomic regions of an average size of 1.7 Mb that contain clusters of highly stringent LXR binding sites and up to 11 T09 target genes. In total we identified 432 T09 target genes within these genomic LXR hotspots. Although each of these regions displays a rather individual arrangement of LXR binding sites in relation to up- and down-regulated genes, they seem to represent the core of the genome-wide activities of LXR. However, 13 of the 112 regions do not have any T09 target gene, suggesting that these stringent LXR locations may have effects on more distant target genes.

In total our microarray analysis of the 4 h T09-stimulated human macrophage-type cells lists 1713 genes, 73% of which are up-regulated, i.e. more than double as many genes are up-regulated than down-regulated. Out of these T09 target genes, 432 (25%) are found within the 112 genomic LXR hotspots, 814 genes (48%) have an LXR binding site within 100 kb distance from the respective gene's TSS and for a further 249 genes (15%) an LXR location within 1 Mb of the TSS could be identified. This rate is similar to other reports comparing ChIP-Seq and microarray results [42]. Nevertheless, this raises the question about the regulation of the 650 T09 target genes without an LXR peak. These genes may be secondary LXR targets. As only 15% of them are down-regulated, a trans-repression mechanism is not very likely.

Our study confirmed a number of known primary LXR target genes, such as *ABCA1*, *ABCG1*, *MYLIP*, *NR1H3 *and *SCD*, but we identified also a number of previously unknown, novel LXR targets. The most up-regulated LXR target gene, *NACA*, encodes for the nascent polypeptide associated complex alpha protein, which is associated to translation and protein folding related processes and when depleted is responsible of triggering endoplasmic reticulum stress-driven apoptosis in hypoxic cells [47]. The *PTGES3 *gene, which is co-located with the *NACA *gene, encodes for the co-chaperone protein prostaglandin E synthase 3 and is required together with the primary chaperone proteins for proper folding and functioning of the glucocorticoid receptor and other steroid receptors [48]. The most down-regulated gene, *SLC3A2*, is related to various processes, for instance in the migration of leukocytes from blood to the central nervous system [49], but also to serum cholesterol levels [50]. Also the *SMPDL3A *gene is an interesting new LXR target, but it has not yet been well characterized as there is only one report linking *SMPDL3A *to bladder tumorigenesis [51].

LXR actions have been related to a number of autoimmune diseases, such as multiple sclerosis [52,53], rheumatoid arthritis [54,55] and experimental autoimmune encephalomyelitis [56]. Interestingly, the association of LXR with autoimmune and metabolic diseases is also one of the major results of the annotation analysis of the 1063 T09 responding genes that we consider as true LXR target genes. The analysis showed the expected relation to lipid metabolism and transport genes, but did not provide any significant link to genes related to innate immunity. However, the LXR regulation of inflammatory cytokines is generally observed in experimental settings, where lipopolysaccharide is used for stimulation and where longer LXR ligand treatment times are allowed [57]. Under these conditions inflammatory cytokines are induced via the transcription factors AP1 and NF-κB and can be inhibited via tethered LXR. In this study, we used PMA for the differentiation of THP-1 cells and T09 treatment of only 4 h. Interestingly, under our experiment conditions, we observed a strong association with apoptosis as more than 70 genes within the 1063 candidates are related to this physiological process. The link of LXRs to apoptosis has is already been reported, not only in macrophages [58], but also in pancreatic β-cells [59] and different cancer cells [60,61].

## Conclusion

We present here the first genome-wide view on LXR locations in a human macrophage-type cellular system. The core action of LXR is focused on 112 genomic hotspots that contain 432 target genes. In total 1063 of the 1713 LXR target genes can be explained by a direct action of LXR, many of which have not been reported before. These genes are related to lipid metabolism and transport and to apoptosis, but not directly to immune functions.

## Methods

### Cell culture

THP-1 human monocytic leukemia cells were grown in RPMI 1640 supplemented with 10% fetal calf serum, 2 mM L-glutamine, 0.1 mg/ml streptomycin and 100 U/ml penicillin and the cells were kept at 37°C in a humidified 95% air/5% CO_2 _incubator. For differentiation into macrophage-type cells the THP-1 cells were incubated for 3 days with 20 nM PMA. Prior to stimulation with 1 μM of the synthetic LXR ligands T09 or GW (both from Sigma-Aldrich) or vehicle (dimethyl sulfoxide (DMSO), final concentration 0.1%) the medium was replaced by RPMI 1640 supplemented with 5% lipid-depleted fetal calf serum, 2 mM L-glutamine, 0.1 mg/ml streptomycin and 100 U/ml penicillin and 20 nM PMA.

### Generation of LXR antibodies

Rat LXRα and LXRβ proteins were purified as previously described [62]. Rabbits were immunized using a standard immunization program at Agrisera (Vännäs, Sweden). In brief, four injections with a total of 0.25 mg of both LXRα and LXRβ protein were performed and serum was collected after 15 weeks. Purified LXRα and LXRβ proteins (1.4 mg) in 0.2 M NaHCO_3_, 0.5 M NaCl, pH 8.3 buffer were coupled on a N-hydroxysuccinimide activated matrix column and the immunized rabbit serum was added to the column, which was washed according to standard procedure. Elution of anti-LXR antibody was performed in 10 cycles and all fractions were pooled.

### ChIP-seq

PMA-differentiated THP-1 cells (per condition nine 175 cm^2 ^flasks, each 2 × 10^7 ^cells, density of 1 × 10^6 ^cells/ml) were treated for 60 min with 1 μM T09 or vehicle (DMSO). Then nuclear proteins were cross-linked to DNA by adding formaldehyde directly to the medium to a final concentration of 1% following incubation for 10 min at room temperature on a rocking platform. Cross-linking was stopped by adding glycine to a final concentration of 0.15 M and incubating at room temperature for 10 min on a rocking platform. The cells were collected by centrifugation and washed twice with ice cold PBS (140 mM NaCl, 2.7 mM KCl, 1.5 mM KH_2_PO_4_, 8.1 mM Na_2_HPO_4_^.^2H_2_O). Nuclear extraction was performed by adding 500 μl PIPES buffer (5 mM Pipes pH 8.0, 85 mM KCl, 0.5% Nonidet P-40, protease inhibitors), incubating for 10 min on ice and removing cytoplasmic components by centrifugation. Nuclear pellets were dissolved in 500 μl SDS lysis buffer (1% SDS, 10 mM EDTA, protease inhibitors, 50 mM Tris-HCl, pH 8.1) and incubated 10 min on ice. Lysates were sonicated at high power with 22 × 30 s pulses in a Bioruptor (Diagenode, Liège, Belgium) to result in DNA fragments of 100 to 600 bp. Cellular debris were removed by centrifugation. Aliquots of 100 μl of the lysate were diluted 1:10 in ChIP dilution buffer (0.01% SDS, 1.1% Triton X-100, 1.2 mM EDTA, 167 mM NaCl, protease inhibitors, 16.7 mM Tris-HCl, pH 8.1) and 2 μg of anti-LXR antibody [20] or non-specific anti-IgG rabbit (sc-2027, Santa Cruz Biotechnologies, Santa Cruz, CA, USA) were added and the samples were incubated for overnight at 4°C on a rotating platform. The immunocomplexes were collected using 60 μl of BSA-coated protein A agarose bead slurry (Millipore) for 3 h at 4°C with rotation. The beads were washed sequentially for 4 min in rotating platform with 1 ml of the following buffers: low salt wash buffer (0.1% SDS, 1% Triton X-100, 2 mM EDTA, 150 mM NaCl, 20 mM Tris-HCl, pH 8.1), high salt wash buffer (0.1% SDS, 1% Triton X-100, 2 mM EDTA, 500 mM NaCl, 20 mM Tris-HCl, pH 8.1) and LiCl wash buffer (0.25 M LiCl, 1% Nonidet P-40, 1% sodium deoxycholate, 1 mM EDTA, 10 mM Tris-HCl, pH 8.1). Finally, the beads were washed twice with 1 ml TE buffer (1 mM EDTA, 10 mM Tris-HCl, pH 8.0) and the immune complexes were eluted twice using 200 μl elution buffer (1% SDS, 100 mM NaHCO_3_) for 15 min at room temperature with rotation. The supernatants were combined and the immune complexes were reverse cross-linked overnight at 65°C in the presence of proteinase K (Fermentas) in a final concentration of 0.1 mg/ml. DNA was extracted with the ChIP DNA Clean & Concentrator Kit (Zymo Research Cooperation, HiSS Diagnostics, Freiburg, Germany) according to manufacturer's instructions and eluted in 40 μl nuclease-free H_2_O. The ChIP templates were sequenced using a Solexa Gene Analyzer II platform (Illumina) at 36 bp read length using standard manufacturer protocols at the Genomics Core Facility in Heidelberg, Germany.

### ChIP-seq data analysis

Some of our following in-house bioinformatics tools were already described recently [41,63]. Alignment of sequence reads produced by T09-treated anti-LXR immunoprecipitated sample, vehicle treated anti-LXR immunoprecipitated sample and the IgG immunoprecipitated negative control sample against the reference genome of version hg19 was done using Bowtie software version 0.12.2 [22]. Command line arguments used with Bowtie were: *bowtie -n 1 -m 1 -e 70 -l 28 -k 1 -t -p 8 -q -S --best hg19 input_file_name output_file_name*. MACS program version 1.3.7.1 [23] was used for finding statistically significant peaks from the alignment sequences using the following arguments: *macs --pvalue = 1e-3 --nomodel --wig -t input_sample_file_name -c input_control_file_name --tsize = 36 --format = BAM --name = analysis_topic --mfold = 13 --shiftsize = 250 --bw = 250 --verbose = 3*. Subsequent refinement of MACS peaks was done using the PeakSplitter program [64] with arguments: *PeakSplitter -p peak_folder_name -w aligned_read_wig_folder_name -o output_folder -c 5 -v 0.6 -f*. An in-house R script was further used to calculate FEs, P-values and FDR estimates for the found subpeaks using an identical approach to MACS 1.3.7.1. Since splitting the original peaks into several subpeaks may produce large sets of weaker flanking residual peaks, distorting for example the genomic and FE distributions of the peaks, we kept for further analysis only those peaks that either had FDR < 1% or were the best subpeak (by FDR) within their parental MACS peak.

Further data analyses were conducted using an in-house R pipeline containing tools for identifying peaks overlapping in two samples, the analysis of genomic distribution of peaks and the integration of peak and gene expression datasets. In the analysis of overlapping peaks, we require that the narrower of the two overlapping peaks shares at least 30% overlap with the broader peak. Under these conditions also any weaker peak (still fulfilling the criterion of raw P < 10^-3 ^reported by the MACS program) in one sample can have FDR < 5%, FDR < 1% or high stringency peak sets, when the overlapping peak in the other sample fulfills the criterion. Since many genomic positions cannot be uniquely assigned to the selected 10 types of genomic elements used in the analysis of peak distributions therein, in the genomic element analysis a prioritization scheme was employed, where the peaks were uniquely overlapped to the elements in a step-wise exclusive scheme, starting from coding elements (UTRs and coding exons), and then moving to introns and outward from the gene.

For comparison with the ChIP-Seq data from a mouse macrophage cell line [18] all peak coordinates from that study were mapped to the human hg19 genome version using Batch Coordinate Conversion tool available at the UCSC Genome Browser [65].

### Motif analysis

*De novo *analysis of LXR binding locations was performed using stand alone version of MEME [24] on sequences within ± 100 bp of the summits of the LXR peaks. Peak sets with FDR < 1% and FDR < 5% with different FE cutoffs FE > 1, FE > 2 etc. from T09- and vehicle-treated samples were analyzed separately in a batch run. The analysis of peak sequences from the T09-treated sample resulted in DR4-type REs in the top 10 of the MEME results with FDR < 1% peaks, when using the cutoff FE > 2 (motif shown in Figure 1B) or higher (tested until FE > 10). DR4-type REs could not be detected, when similar *de novo *analysis for the peak sequences obtained from the vehicle-treated sample or all peak sequences with FDR < 5% from the T09-treated sample were performed.

Identification of DR4-type REs within LXR peak regions was performed using the RSAT matrix scan tool available at http://rsat.ulb.ac.be/rsat[26]. Two matrices were used as a model for a DR4-type RE: the *de novo *detected matrix (Figure 1B) and the same matrix modified from the positions 7 and 8 within the spacer to resemble more the DR4-type RE known in the literature. The modification was made by setting at these positions the frequency of any nucleotide equal (3). The analysis of JASPAR matrices (JASPAR core, version 10/2009) was performed in similar manner. For the background model, the input peak sequences were used to take into account the nucleotide content within these regions. Also for the analysis of DR-, ER- and IR-type REs the RSAT dna-pattern tool was used. One mismatch was allowed for an individual nuclear receptor binding site.

### qPCR

Total RNA was extracted using High Pure RNA Isolation kit (Roche). cDNA synthesis was performed using Transcriptor First Strand cDNA Synthesis Kit (Roche), using 1 μg of total RNA as a template and 50 pmol oligo(dT)_18 _primers. qPCR was performed using a LightCycler^® ^480 System (Roche). The reactions were performed using 4 pmol of reverse and forward primers, 4 μl cDNA template and FastStart SYBR Green Master (Roche) in a total volume of 10 μl. In the PCR reaction the DNA templates were pre-denaturated at for 10 min at 95°C, followed by amplification steps cycles of 20 s denaturation at 95°C, 15 s annealing at primer specific temperatures (see additional file 10: **Table S5**), 15 s elongation at 72°C and a final elongation for 10 min at 72°C. PCR product quality was monitored using post-PCR melt curve analysis. Fold inductions were calculated using the formula 2^-(ΔΔCt)^, where ΔΔCt is ΔCt(stimulus)-ΔCt(solvent), ΔCt is Ct(target gene)-Ct(contol gene) and the Ct is the cycle, at which the threshold is crossed. Relative expression levels of the target genes were normalized to the internal control gene *RPLP0*.

### Microarray analysis

Total RNA was checked for RNA integrity using a Biorad Experion automated electrophoresis system (Biorad, Nazareth, Belgium). None of the RNA samples showed any sign of degradation. Triplicate samples were analyzed with HumanHT-12 v3 Expression BeadChips from Illumina (San Diego, CA, USA) at the Finnish Microarray Centre (Turku, Finland). Raw data are available at GEO under accession GSE28319. Analyses of microarray data were performed using R statistical software version 2.11 [66] with associated libraries from Bioconductor project version 2.6 [67]. Data were normalized using VST transformation and RSN normalization used as standard approach for Illumina arrays. Normalized data were filtered in order to remove probes without detected signal for any of the samples. Probe sets that were not linked to any known or predicted human gene were also filtered out. Linear Models for Microarray Data (limma) package [68] using linear model fitting for statistical testing with empirical Bayes variance smoothing procedure was applied to detection of differentially expressed genes. Obtained P-values were corrected for multiple testing using the Benjamini & Hochberg FDR procedure [69]. For downstream analysis, GO [70] biological process terms were tested for enrichment.

### Spatial clustering of LXR binding locations

For the analysis of spatial clusters of LXR binding locations we first performed a density analysis of genomic coordinates of LXR peak summit locations in T09- and vehicle-treated samples and the starting coordinates of up- and down-regulated genes. This was made using the standard R function "density" with a 1 Mb size for the sliding window with 0.5 Mb steps over each chromosome using the default Gaussian window kernel function. The density values resulting from each of these windows were weighted using FE values for LXR binding locations or logarithmic FCs between T09- and vehicle-treated samples for the locations of regulated genes. In order to detect the exact borders of the LXR hotspot regions, we performed a subsequent clustering of the density data representing the LXR binding locations by using methods developed originally for the analysis of array CGH data implemented in R package SegClust [71,72]. First, the density vectors representing LXR binding site distributions in T09- and vehicle-treated samples were combined by taking the maximum value for the two samples in each genomic location. The resulting combined density data were used for the "segmean" function, implemented in SegClust, performing a dynamic programming-based algorithm for finding the optimal breakpoints in terms of changes of mean for a fixed number of breakpoints *K *[72]. The method was applied using various *K *starting from 1 ending to the number of analyzed data points. The results were further used for the "segselect" function, which uses an adaptive model selection [71] between outcomes with different numbers of breakpoints *K*. A result optimal in terms of this model selection was chosen as representative for each chromosome. The resulting chromosomal regions were selected for containing at least one LXR binding location within the high stringency set of peaks resulting in 112 separate regions. The region borders were widened 100 kb upstream of the 5'-border and 100 kb downstream of the 3'-border of each region to cover putative target genes in these regions.

## Abbreviations

ABC: ATP-binding cassette transporter; ACLS3: acyl-CoA synthetase long-chain family member 3; ADM: adrenomedullin; ChIP: chromatin immunoprecipitation; CNNM4: cyclin M4; DE: differentially expressed; DMSO: dimethyl sulfoxide; DR4: direct repeat spaced by 4 nucleotides; EGR1: early growth response protein; ER: everted repeat; ETF1: eukaryotic translation termination factor 1; EWSR1: Ewing sarcoma breakpoint region 1; FDR: false discovery rate; FLI1: friend leukemia virus integration 1; FE: fold enrichment; GPR137: G protein-coupled receptor 137; GO: gene ontology; GW: GW3965; HARS: histidyl-tRNA synthetase; IgG: immunoglobulin gamma; IR: inverted repeat; KLF4: krüppel-like factor 4; LXR: liver X receptor; MYF: myogenic factor; MYLIP: myosin regulatory light chain interact; NACA: nascent polypeptide-associated complex alpha subunit; NR1H3: LXRα; PMA: phorbol myristate acetate; PPAR: peroxisome proliferator-activated receptor; PRDX5: peroxiredoxin 5, PRIM1, primase, DNA, polypeptide 1; PTGES3: prostaglandin E synthase 3; PUF60: poly-U binding splicing factor 60 KDa; PWM: position weight matrix; qPCR: quantitative real-time PCR; RE: response element; RPLP0: ribosomal protein large P0; RREB1: Ras-responsive element-binding protein 1; RSAT: regulatory sequence analysis tools; RXR: retinoid X receptor; SCD: stearoyl-CoA desaturase; SCL3A2: solute carrier family 3A2; SMPDL3A: sphingomyelin phosphodiesterase: acid-like 3A; SP1: Sp1 transcription factor; SPIB: Spi-B transcription factor; T09: T0901317; TATDN2: TatD DNase domain containing 2; TRMT112: tRNA methyltransferase 11-2 homolog; TSS: transcription start site; VDR: vitamin D receptor.

## Authors' contributions

PP, LW-S and CC designed the project, LW-S, JD and JR performed the experiments, PP, AW-B and SH analyzed the data, ET and KRS provided material and participated in the discussion and PP and CC wrote the manuscript. All authors read and approved the final manuscript.

## Supplementary Material

Additional file 1 **Figure S1. Validation of LXR antibody**. Protein extracts from livers of WT, LXRα^-/-^, LXRβ^-/- ^and LXRαβ^-/- ^mice [73] (**A**) or from HeLa cells, which were transfected with the empty pSG5 expression vector as control and pSG5 expressing human LXRα or LXRβ, respectively, using FuGENE 6 transfection reagent (Roche) (**B**), were separated on 8% sodium dodecyl sulfate (SDS) polyacrylamide gels. Western blotting using anti-LXR antibody was then performed using standard procedures. Click here for file

Additional file 2 **Table S1. LXR binding locations in a human macrophage-type cell line**. Columns indicate the chromosome, start and end locations of ChIP-Seq peaks, peak length and summit, P-values from Poisson distribution, FE in comparison to IgG, FDR value and information on which sample peak was detected (T09- or vehicle-treated). Information regarding to any peak present in both samples is presented in two separate rows. Click here for file

Additional file 3 **Figure S2. Distribution of genomic LXR binding sites to genomic elements**. **A **Distribution of LXR binding locations from the high stringent set of peaks to different genomic elements. Unique peaks represent binding locations present only in one of the two samples (disjoint areas of Venn diagram in Figure 1A) and in both samples (joint set of Venn diagram in Figure 1A). **B **Distribution of peaks around TSSs of closest genes. Click here for file

Additional file 4 **Figure S3. RE types present within the 202 high stringency LXR peak set**. Proportions of high stringency set of peaks containing direct repeats (DRs), everted repeats (ERs) and inverted repeats (IRs). Search has been made with RSAT DNA-pattern tool [26] using RGKTCA half-site with indicated number of spacings. Click here for file

Additional file 5 **Table S2. Differentially expressed genes in a human macrophage-type cell line**. Columns indicate the chromosome, start of the T09 target gene, log2 expression after T09- and vehicle-treatment, adjusted P-value for gene expression difference, gene name and FC. The detected LXR binding locations within proximal (± 100 kb) and distal (± 1 Mb) area from the respective gene TSS are displayed on the right side of the table. Click here for file

Additional file 6 **Table S3. Detailed information on all 112 LXR hotspots**. Numbers of peaks and genes are indicated similarly as in Table 1. Additional columns indicate the tendency of each region having vast majority (> 2 times) of peaks in T09- (or vehicle-) treated sample versus vehicle- (or T09-) treated sample, the tendency of each region having majority (> 2 times) of up- (or down-) regulated genes versus down- (up-) regulated genes. Moreover, in separate columns up- and down-regulated genes, number of all genes, gene density and enriched GO terms (P < 0.05) are shown. Regions already shown in Table 1 are in bold. Click here for file

Additional file 7 **Figure S4. Enrichment statistics of LXR binding locations**. The number of LXR peaks in one of the 112 hotspot regions (Figure 2) is compared with the number of all genes (**A**), the number of DE genes with adjusted P < 0.01 (**B**), the proportion of DE genes (**C**) and the density of DE genes (**D**). Click here for file

Additional file 8 **Figure S5. LXR target gene validation**. PMA-differentiated THP-1 cells were treated for 4 h with vehicle (DMSO), 1 μM GW3965 (GW) or 1 μM T0901317 (T09), total RNA was extracted and qPCR was performed with primers specific for selected genes. The data were normalized to the expression of the housekeeping gene *RPLP0 *and fold inductions were calculated in reference to vehicle control. Columns indicate the means of four independent cell treatments and the bars represent standard deviations. Student's t-test was performed to determine the significance of the stimulation in reference to vehicle-treated control (** P < 0.01; *** P < 0.001). Click here for file

Additional file 9 **Table S4. Enrichment analysis for all LXR target genes**. The analysis was performed using the DAVID tool [37] for all 1063 LXR target genes with P < 0.01 for differential expression. The columns indicate GO identifier, term name, count of associated target genes and the P-value and FDR for the enrichment. Click here for file

Additional file 10 **Table S5. qPCR primers used in the validation of gene expression microarray results of select genes**. Click here for file
