# Supplementary figures and images for: Genome-wide landscape of liver X receptor chromatin binding and gene regulation in human macrophages
Source: BMC Genomics. 2012 Jan 31;13:50. doi: 10.1186/1471-2164-13-50 (PMC3295715; doi:10.1186/1471-2164-13-50)

# Figure S1

**A**

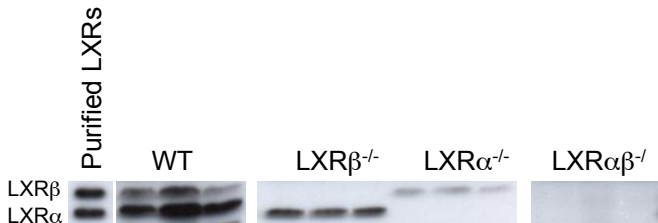

**B**

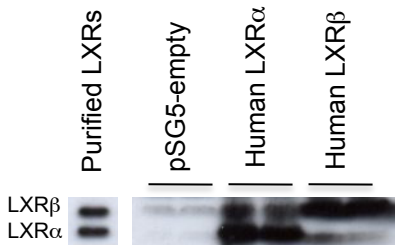

Supplement: Additional file 1 — Figure S1. Validation of LXR antibody. Protein extracts from livers of WT, LXRα-/-, LXRβ-/- and LXRαβ-/- mice [73] (A) or from HeLa cells, which were transfected with the empty pSG5 expression vector as control and pSG5 expressing human LXRα or LXRβ, respectively, using FuGENE 6 transfection reagent (Roche) (B), were separated on 8% sodium dodecyl sulfate (SDS) polyacrylamide gels. Western blotting using anti-LXR antibody was then performed using standard procedures. [file 1471-2164-13-50-S1.PDF]

**Figure S2**

**A**

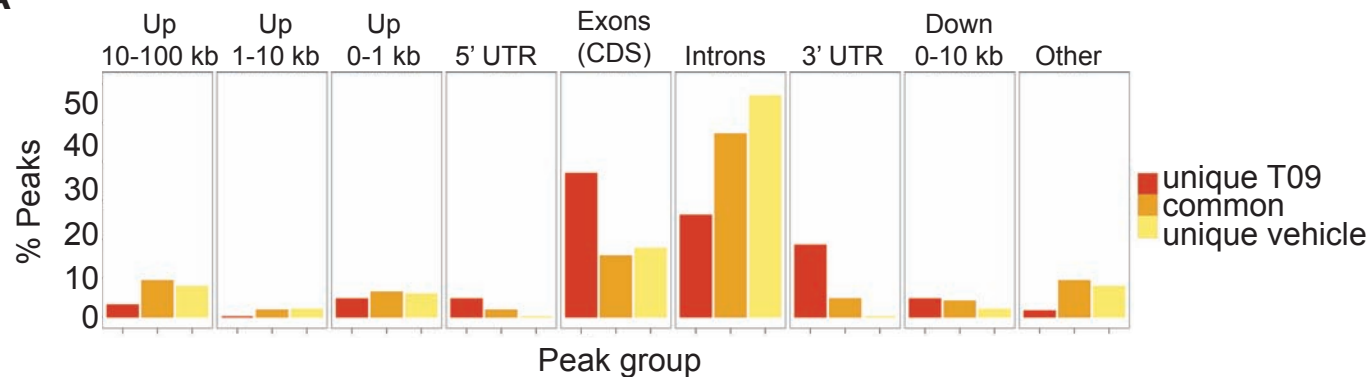

**B**

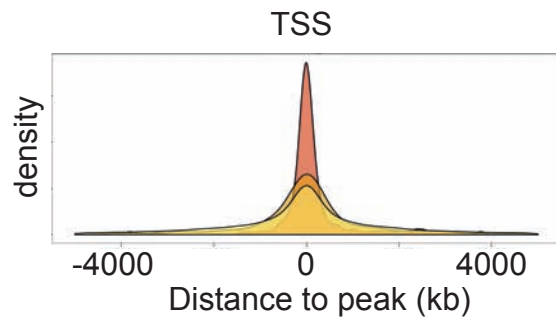

Supplement: Additional file 3 — Figure S2. Distribution of genomic LXR binding sites to genomic elements. A Distribution of LXR binding locations from the high stringent set of peaks to different genomic elements. Unique peaks represent binding locations present only in one of the two samples (disjoint areas of Venn diagram in Figure 1A) and in both samples (joint set of Venn diagram in Figure 1A). B Distribution of peaks around TSSs of closest genes. [file 1471-2164-13-50-S3.PDF]

Figure S3

Direct repeats

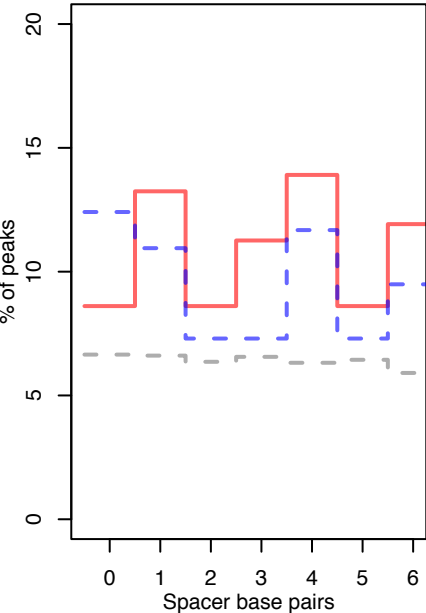

Everted repeats

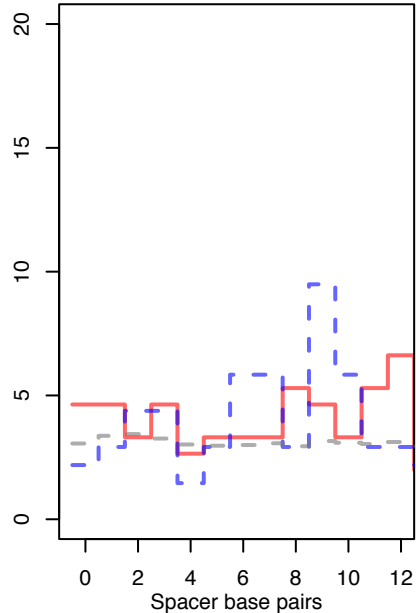

Inverted repeats

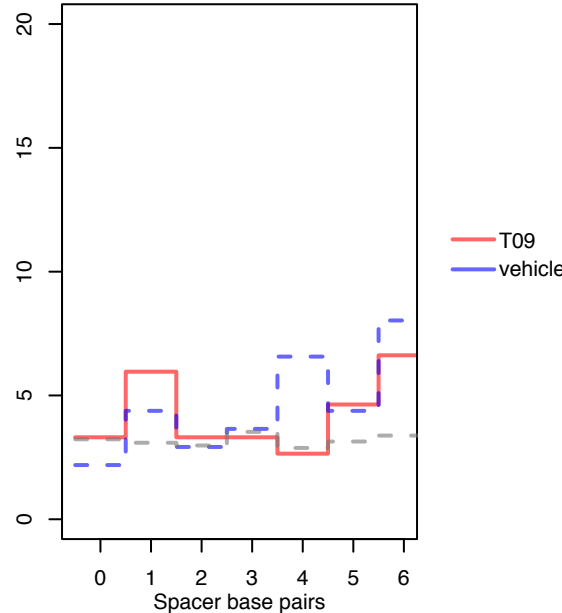

Supplement: Additional file 4 — Figure S3. RE types present within the 202 high stringency LXR peak set. Proportions of high stringency set of peaks containing direct repeats (DRs), everted repeats (ERs) and inverted repeats (IRs). Search has been made with RSAT DNA-pattern tool [26] using RGKTCA half-site with indicated number of spacings. [file 1471-2164-13-50-S4.PDF]

**Figure S4**

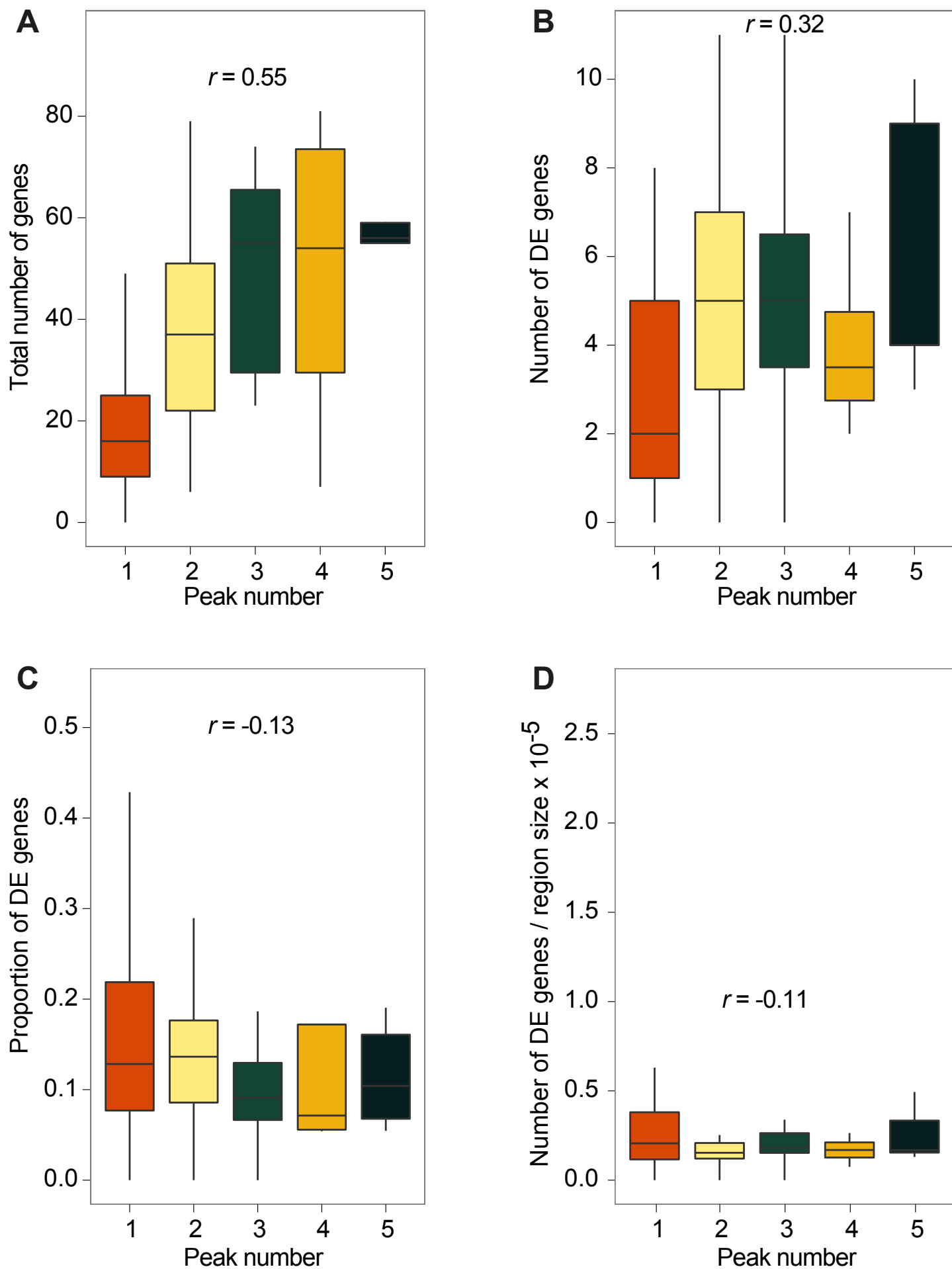

Supplement: Additional file 7 — Figure S4. Enrichment statistics of LXR binding locations. The number of LXR peaks in one of the 112 hotspot regions (Figure 2) is compared with the number of all genes (A), the number of DE genes with adjusted P < 0.01 (B), the proportion of DE genes (C) and the density of DE genes (D). [file 1471-2164-13-50-S7.PDF]

**Figure S5**

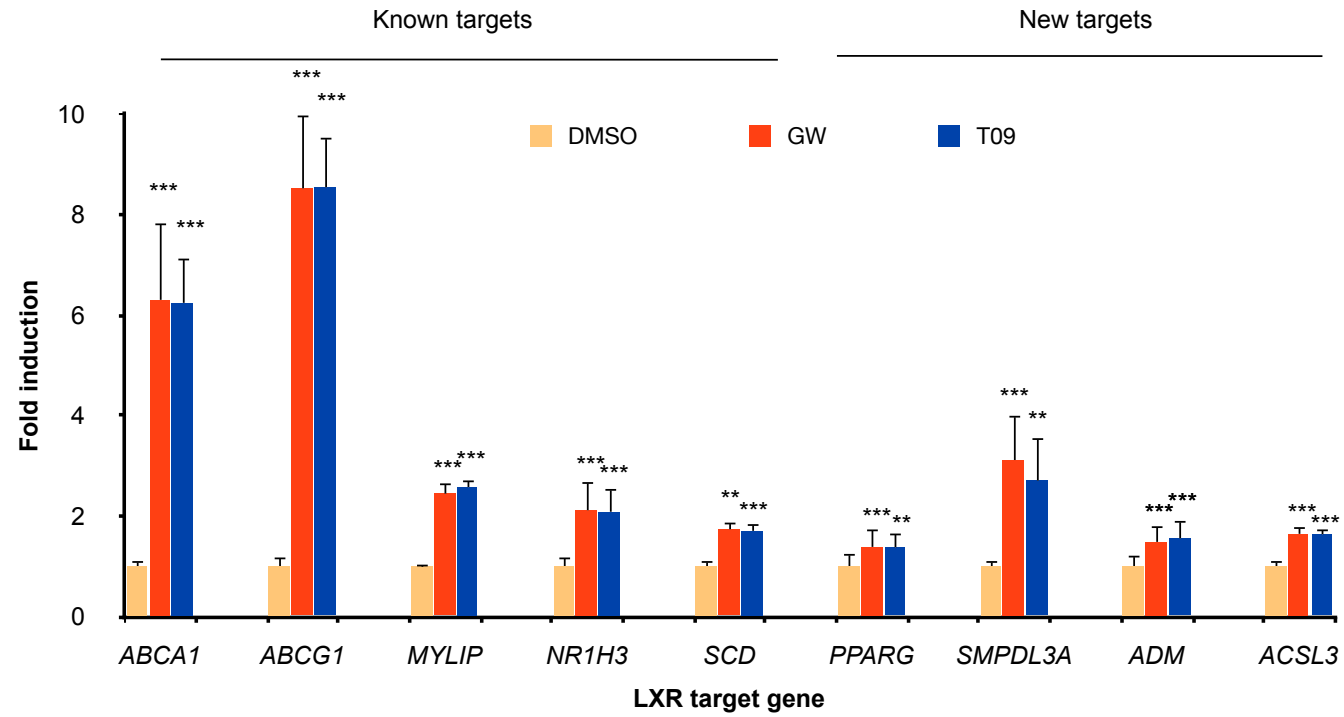

Supplement: Additional file 8 — Figure S5. LXR target gene validation. PMA-differentiated THP-1 cells were treated for 4 h with vehicle (DMSO), 1 μM GW3965 (GW) or 1 μM T0901317 (T09), total RNA was extracted and qPCR was performed with primers specific for selected genes. The data were normalized to the expression of the housekeeping gene RPLP0 and fold inductions were calculated in reference to vehicle control. Columns indicate the means of four independent cell treatments and the bars represent standard deviations. Student's t-test was performed to determine the significance of the stimulation in reference to vehicle-treated control (** P < 0.01; *** P < 0.001). [file 1471-2164-13-50-S8.PDF]
