# Supplementary material for: Genome-wide landscape of liver X receptor chromatin binding and gene regulation in human macrophages
Source: BMC Genomics. 2012 Jan 31;13:50. doi: 10.1186/1471-2164-13-50 (PMC3295715; doi:10.1186/1471-2164-13-50)
Supplement: Additional file 10 — Table S5. qPCR primers used in the validation of gene expression microarray results of select genes. [file 1471-2164-13-50-S10.PDF]

Table S5. qPCR primers used in the validation of gene expression microarray results of select genes.

Gene	Fragment size (bp)	Annealing temperature (°C)	Primer sequences (5’-3’)
ABCA1	88	58	CTACCCACCCTATGAACAAC<br>GATAATCCCCTGAACCCAAG
ABCG1	145	62	AGGGATTTGGGTCTGAACTG<br>GGTCTCTCTTGTGGTCTGAG
MYLIP	67	60	CACGCATTCTACAGGTGTG<br>CCTTCAAGTCACGGCTATAC
NR1H3	67	58	GAGTGTGTCCTGTCAGAAG<br>GAGCCTGTTCCTCCTCTTG
PPARG	409	60	CGACCAAGTAACTCTCCTCA<br>GTTCCGTGACAATCTGTCTG
SMPDL3A	166	60	AGTAGCAAACCTCTGGAAAC<br>GTCAGTCTTGTTCAGTGTC
SCD	115	60	GTGGGTTGGCTGCTTGTG<br>GTTTGTAGTACCTCCTCTGG
ADM	105	58	GAATAAGTGGGCTCTGAGTC<br>CGAATAAGGGTCTGGGCAG
ACSL3	130	60	CGGCACATCATCACTGTTG<br>TGCTATGAGGTTGGTTTTCC
RPLP0	318	58-62	AGATGCAGCAGATCCGCAT<br>GTGGTGATACCTAAAGCCTG
